# Crystal structure of 3-{1-[(1-allyl-1*H*-indazol-6-yl)amino]­ethyl­idene}-6-methyl-2*H*-pyran-2,4(3*H*)-dione

**DOI:** 10.1107/S1600536814024520

**Published:** 2014-11-15

**Authors:** Mohamed El Ghozlani, El Mostapha Rakib, Ahmed Gamouh, Mohamed Saadi, Lahcen El Ammari

**Affiliations:** a Laboratoire de Chimie Organique et Analytique, Université Sultan Moulay Slimane, Faculté des Sciences et Techniques, Béni-Mellal, BP 523, Morocco; b Laboratoire de Chimie du Solide Appliquée, Faculté des Sciences, Université Mohammed V-Agdal, Avenue Ibn Battouta, BP 1014, Rabat, Morocco

**Keywords:** crystal structure, pyran-2,4-dione, pharmacological activity, indazole derivatives

## Abstract

In the title compound, C_18_H_17_N_3_O_3_, the dihedral angle between the planes of the indazole ring system [maximum deviation = 0.012 (1) Å] and the pyran-2,4-dione ring is 54.03 (6)°. An intra­molecular N—H⋯O hydrogen bond closes an *S*(6) ring. The same H atom also participates in an inter­molecular N—H⋯O hydrogen bond, which generates an inversion dimer. The dimers are linked by weak C—H⋯O contacts, thereby forming a three-dimensional network.

## Related literature   

For pharmacological activities of indazole derivatives, see: Cerecetto *et al.* (2005 ▶); Jennings & Tennant (2007 ▶); Sun *et al.* (1997 ▶). For innovative methods in their synthesis, see: Paul *et al.* (2014 ▶).
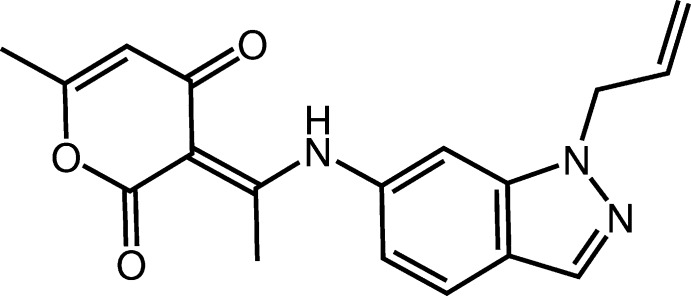


## Experimental   

### Crystal data  

C_18_H_17_N_3_O_3_
*M* *_r_* = 323.35
Triclinic, 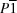

*a* = 6.7708 (11) Å
*b* = 10.5761 (17) Å
*c* = 11.9643 (17) Å
α = 88.239 (9)°
β = 81.123 (9)°
γ = 79.140 (9)°
*V* = 831.3 (2) Å^3^
*Z* = 2
Mo *K*α radiation
μ = 0.09 mm^−1^
*T* = 296 K
0.39 × 0.34 × 0.29 mm

### Data collection  

Bruker X8 APEX CCD diffractometer
Absorption correction: multi-scan (*SADABS*; Bruker, 2009 ▶) *T*_min_ = 0.685, *T*_max_ = 0.746
11752 measured reflections
3929 independent reflections
2930 reflections with *I* > 2σ(*I*)
*R*_int_ = 0.027

### Refinement  

*R*[*F* ^2^ > 2σ(*F* ^2^)] = 0.044
*wR*(*F* ^2^) = 0.131
*S* = 1.04
3929 reflections
217 parameters
H-atom parameters constrained
Δρ_max_ = 0.19 e Å^−3^
Δρ_min_ = −0.19 e Å^−3^

### 

Data collection: *APEX2* (Bruker, 2009 ▶); cell refinement: *SAINT* (Bruker, 2009 ▶); data reduction: *SAINT*; program(s) used to solve structure: *SHELXS97* (Sheldrick, 2008 ▶); program(s) used to refine structure: *SHELXL97* (Sheldrick, 2008 ▶); molecular graphics: *ORTEP-3 for Windows* (Farrugia, 2012 ▶); software used to prepare material for publication: *PLATON* (Spek, 2009 ▶) and *publCIF* (Westrip, 2010 ▶).

## Supplementary Material

Crystal structure: contains datablock(s) I. DOI: 10.1107/S1600536814024520/hb7311sup1.cif

Structure factors: contains datablock(s) I. DOI: 10.1107/S1600536814024520/hb7311Isup2.hkl

Supporting information file. DOI: 10.1107/S1600536814024520/hb7311Isup3.cml

**Fig. 1.**
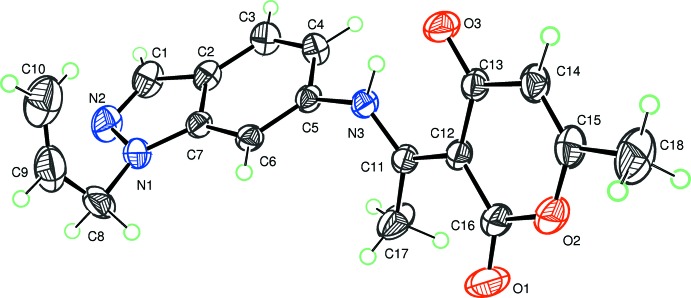
Molecular structure of the title compound with displacement ellipsoids drawn at the 50% probability level.

. DOI: 10.1107/S1600536814024520/hb7311fig1.tif
Mol­ecular structure of the title compound with displacement ellipsoids drawn at the 50% probability level.

**Fig. 2.**
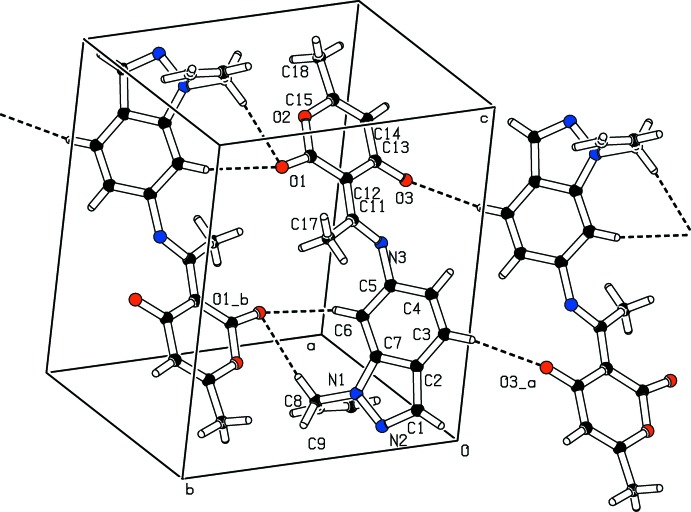
Partial crystal packing for the title compound showing N3—H3N···O3, C6—H6···O1 and C8—H8A···O1 hydrogen bonds as dashed lines.

N A . DOI: 10.1107/S1600536814024520/hb7311fig2.tif
Partial crystal packing for the title compound showing N3—H3*N*⋯O3, C6—H6⋯O1 and C8—H8*A*⋯O1 hydrogen bonds as dashed lines.

CCDC reference: 1033266

Additional supporting information:  crystallographic information; 3D view; checkCIF report

## Figures and Tables

**Table 1:** Hydrogen-bond geometry (, )

*D*H*A*	*D*H	H*A*	*D* *A*	*D*H*A*
N3H3*N*O3	0.91	1.77	2.5567(15)	143
N3H3*N*O3^i^	0.91	2.51	3.0612(16)	120
C6H6O1^ii^	0.93	2.54	3.3554(19)	146
C8H8*A*O1^ii^	0.97	2.53	3.495(2)	173

Symmetry codes: (i) 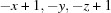; (ii) 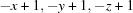.

## supplementary crystallographic information

### S1. Comment

Pharmacologically active indazole and its derivatives are widely used as drug in treating various human diseases including cancer, inflammation, cardiovascular, and others (Cerecetto, *et al.*, 2005; Jennings & Tennant, 2007; Sun, *et al.*, 1997). This has incited researchers to develop innovative methods in their synthesis (Paul, *et al.*, 2014).

The two fused five- and six-membered rings (N1/N2/C1 to C7), part of the molecule of the title compound, are almost planar, with the maximum deviation of 0.012 (1) Å arising from atom N1. The fused rings system is nearly perpendicular to the allyl group (C8 C9 C10) as shown in Fig. 1 (torsion angle C9 C8 N1 C7 = 88.9 (2)°). Moreover, the dihedral angle between the indazole system and the plan through the atoms forming the pyran-2 ring (C12 to C16O) is 54.03 (6)°.

In the crystal, molecules are connected through N—H···O hydrogen bonds in the way to build dimers which are linked by weak C—H···O contacts, forming a three-dimensional network as shown in Fig.2 and Table 2.

### S2. Experimental

1-Allyl-6-nitroindazole (1.0 mmol) was added to a mixture of indium powder (450 mg, 3.91 mmol), and acetic acid (1.12 ml, 10 mmol) in THF (2 ml), followed by the addition of 4-hydroxy-6-methyl-2-pyrone (1.0 mmol) in THF (3 ml). The reaction mixture was stirred at 353 K. After the reaction was completed, the reaction mixture was diluted with ethyl acetate (30 ml), filtered through Celite, poured into 10% K_2_CO_3_ (25 ml), and then extracted with ethyl acetate (30 ml *x* 3). The combined organic extracts were dried over MgSO_4_, filtered, and concentrated. The resulting residue was purified by flash chromatography (eluted with Ethyl acetate: Hexane 4:6). The title compound was recrystallized from the solvent mixture ethyl acetate/hexane to yield yellow blocks (yield: 85%, m.p.: 392 K).

### S3. Refinement

H atoms were located in a difference map and treated as riding with C–H = 0.96 Å, C–H = 0.97 Å, C–H = 0.93 Å, and N–H = 0.90 Å for methyl, methylene, aromatic CH and NH respectively. All hydrogen with *U*_iso_(H) = 1.2 *U*_eq_ (methylene,aromatic, NH) and *U*_iso_(H) = 1.5 *U*_eq_ for methyl.

### Crystal data

**Table:**

C_18_H_17_N_3_O_3_	*Z* = 2
*M**_r_* = 323.35	*F*(000) = 340
Triclinic, *P*1	*D*_x_ = 1.292 Mg m^−^^3^
Hall symbol: -P 1	Melting point: 392 K
*a* = 6.7708 (11) Å	Mo *K*α radiation, λ = 0.71073 Å
*b* = 10.5761 (17) Å	Cell parameters from 3929 reflections
*c* = 11.9643 (17) Å	θ = 2.6–27.9°
α = 88.239 (9)°	µ = 0.09 mm^−^^1^
β = 81.123 (9)°	*T* = 296 K
γ = 79.140 (9)°	Block, yellow
*V* = 831.3 (2) Å^3^	0.39 × 0.34 × 0.29 mm

### Data collection

**Table:**

Bruker X8 APEX CCD diffractometer	3929 independent reflections
Radiation source: fine-focus sealed tube	2930 reflections with *I* > 2σ(*I*)
Graphite monochromator	*R*_int_ = 0.027
φ and ω scans	θ_max_ = 27.9°, θ_min_ = 2.6°
Absorption correction: multi-scan (*SADABS*; Bruker, 2009)	*h* = −8→8
*T*_min_ = 0.685, *T*_max_ = 0.746	*k* = −12→13
11752 measured reflections	*l* = −15→15

### Refinement

**Table:**

Refinement on *F*^2^	Primary atom site location: structure-invariant direct methods
Least-squares matrix: full	Secondary atom site location: difference Fourier map
*R*[*F*^2^ > 2σ(*F*^2^)] = 0.044	Hydrogen site location: inferred from neighbouring sites
*wR*(*F*^2^) = 0.131	H-atom parameters constrained
*S* = 1.04	*w* = 1/[σ^2^(*F*_o_^2^) + (0.0634*P*)^2^ + 0.1441*P*] where *P* = (*F*_o_^2^ + 2*F*_c_^2^)/3
3929 reflections	(Δ/σ)_max_ < 0.001
217 parameters	Δρ_max_ = 0.19 e Å^−^^3^
0 restraints	Δρ_min_ = −0.19 e Å^−^^3^

### Special details

**Table:**

Geometry. All e.s.d.'s (except the e.s.d. in the dihedral angle between two l.s. planes) are estimated using the full covariance matrix. The cell e.s.d.'s are taken into account individually in the estimation of e.s.d.'s in distances, angles and torsion angles; correlations between e.s.d.'s in cell parameters are only used when they are defined by crystal symmetry. An approximate (isotropic) treatment of cell e.s.d.'s is used for estimating e.s.d.'s involving l.s. planes.
Refinement. Refinement of *F*^2^ against all reflections. The weighted *R*-factor *wR* and goodness of fit *S* are based on *F*^2^, conventional *R*-factors *R* are based on *F*, with *F* set to zero for negative *F*^2^. The threshold expression of *F*^2^ > σ(*F*^2^) is used only for calculating *R*-factors(gt) *etc*. and is not relevant to the choice of reflections for refinement. *R*-factors based on *F*^2^ are statistically about twice as large as those based on *F*, and *R*- factors based on all data will be even larger.

### Fractional atomic coordinates and isotropic or equivalent isotropic displacement parameters (Å^2^)

**Table:**

	*x*	*y*	*z*	*U*_iso_*/*U*_eq_	
C1	−0.2126 (2)	0.27561 (17)	0.19077 (14)	0.0535 (4)	
H1	−0.3315	0.2495	0.1792	0.064*	
C2	−0.1187 (2)	0.24555 (13)	0.28909 (11)	0.0394 (3)	
C3	−0.1571 (2)	0.17674 (15)	0.38933 (12)	0.0453 (4)	
H3	−0.2704	0.1375	0.4040	0.054*	
C4	−0.0230 (2)	0.16880 (14)	0.46546 (11)	0.0419 (3)	
H4	−0.0454	0.1230	0.5324	0.050*	
C5	0.14799 (19)	0.22876 (12)	0.44391 (10)	0.0327 (3)	
C6	0.1916 (2)	0.29707 (12)	0.34631 (10)	0.0347 (3)	
H6	0.3054	0.3358	0.3323	0.042*	
C7	0.0533 (2)	0.30446 (12)	0.26930 (10)	0.0352 (3)	
C8	0.2094 (3)	0.42381 (18)	0.10121 (14)	0.0664 (5)	
H8A	0.2818	0.4594	0.1529	0.080*	
H8B	0.1464	0.4943	0.0572	0.080*	
C9	0.3587 (4)	0.3318 (3)	0.02309 (16)	0.0820 (7)	
H9	0.4607	0.3652	−0.0227	0.098*	
C10	0.3598 (3)	0.2112 (3)	0.01307 (18)	0.0866 (7)	
H10A	0.2609	0.1733	0.0570	0.104*	
H10B	0.4595	0.1616	−0.0382	0.104*	
C11	0.3460 (2)	0.29146 (12)	0.58397 (10)	0.0349 (3)	
C12	0.4993 (2)	0.24657 (12)	0.65330 (10)	0.0341 (3)	
C13	0.5675 (2)	0.11088 (13)	0.66967 (10)	0.0359 (3)	
C14	0.7230 (2)	0.07406 (14)	0.74087 (11)	0.0429 (3)	
H14	0.7646	−0.0126	0.7568	0.051*	
C15	0.8078 (2)	0.16072 (16)	0.78399 (12)	0.0474 (4)	
C16	0.5936 (3)	0.33761 (15)	0.70210 (12)	0.0482 (4)	
C17	0.2422 (3)	0.42837 (15)	0.57937 (16)	0.0671 (6)	
H17A	0.1140	0.4326	0.5532	0.101*	
H17B	0.3260	0.4753	0.5284	0.101*	
H17C	0.2197	0.4655	0.6535	0.101*	
C18	0.9761 (3)	0.1362 (2)	0.85373 (18)	0.0806 (6)	
H18A	0.9316	0.1796	0.9250	0.121*	
H18C	1.0918	0.1679	0.8145	0.121*	
H18B	1.0133	0.0454	0.8665	0.121*	
N1	0.0511 (2)	0.36448 (12)	0.16653 (10)	0.0472 (3)	
N2	−0.1112 (2)	0.34456 (14)	0.11844 (11)	0.0574 (4)	
N3	0.28794 (17)	0.20639 (10)	0.52426 (9)	0.0350 (3)	
H3N	0.3443	0.1235	0.5379	0.042*	
O1	0.5633 (3)	0.45366 (11)	0.69316 (13)	0.0832 (5)	
O2	0.74794 (18)	0.28942 (11)	0.76619 (9)	0.0567 (3)	
O3	0.50060 (18)	0.02513 (9)	0.62444 (9)	0.0538 (3)	

### Atomic displacement parameters (Å^2^)

**Table:**

	*U*^11^	*U*^22^	*U*^33^	*U*^12^	*U*^13^	*U*^23^
C1	0.0429 (9)	0.0691 (11)	0.0520 (9)	−0.0058 (8)	−0.0233 (7)	−0.0044 (8)
C2	0.0330 (7)	0.0437 (8)	0.0413 (7)	−0.0003 (6)	−0.0124 (5)	−0.0057 (6)
C3	0.0325 (7)	0.0595 (9)	0.0459 (8)	−0.0136 (6)	−0.0057 (6)	−0.0009 (7)
C4	0.0404 (8)	0.0494 (8)	0.0360 (7)	−0.0096 (6)	−0.0055 (6)	0.0045 (6)
C5	0.0351 (7)	0.0320 (6)	0.0313 (6)	−0.0016 (5)	−0.0108 (5)	−0.0025 (5)
C6	0.0392 (7)	0.0317 (6)	0.0362 (6)	−0.0083 (5)	−0.0128 (5)	0.0005 (5)
C7	0.0413 (7)	0.0306 (6)	0.0340 (6)	−0.0015 (5)	−0.0130 (5)	−0.0012 (5)
C8	0.1033 (15)	0.0663 (11)	0.0490 (9)	−0.0470 (11)	−0.0384 (10)	0.0265 (8)
C9	0.0885 (15)	0.130 (2)	0.0434 (9)	−0.0647 (15)	−0.0069 (10)	0.0047 (11)
C10	0.0708 (14)	0.125 (2)	0.0651 (12)	−0.0253 (14)	0.0001 (10)	−0.0205 (13)
C11	0.0409 (7)	0.0328 (7)	0.0307 (6)	−0.0022 (5)	−0.0090 (5)	−0.0023 (5)
C12	0.0401 (7)	0.0347 (7)	0.0288 (6)	−0.0061 (5)	−0.0099 (5)	−0.0020 (5)
C13	0.0405 (7)	0.0375 (7)	0.0298 (6)	−0.0037 (6)	−0.0102 (5)	−0.0011 (5)
C14	0.0483 (8)	0.0446 (8)	0.0344 (6)	0.0024 (6)	−0.0160 (6)	0.0005 (6)
C15	0.0472 (9)	0.0594 (9)	0.0366 (7)	−0.0040 (7)	−0.0163 (6)	−0.0006 (6)
C16	0.0641 (10)	0.0441 (8)	0.0438 (8)	−0.0155 (7)	−0.0247 (7)	0.0010 (6)
C17	0.0909 (14)	0.0388 (8)	0.0736 (11)	0.0151 (8)	−0.0484 (10)	−0.0163 (8)
C18	0.0723 (13)	0.1046 (16)	0.0736 (12)	−0.0078 (12)	−0.0479 (11)	−0.0039 (11)
N1	0.0629 (8)	0.0466 (7)	0.0385 (6)	−0.0134 (6)	−0.0258 (6)	0.0080 (5)
N2	0.0622 (9)	0.0659 (9)	0.0493 (7)	−0.0067 (7)	−0.0324 (7)	0.0040 (6)
N3	0.0408 (6)	0.0308 (5)	0.0347 (5)	−0.0014 (5)	−0.0159 (5)	0.0006 (4)
O1	0.1312 (13)	0.0405 (7)	0.1001 (10)	−0.0286 (7)	−0.0724 (10)	0.0073 (6)
O2	0.0669 (7)	0.0579 (7)	0.0567 (6)	−0.0191 (6)	−0.0349 (6)	−0.0013 (5)
O3	0.0747 (8)	0.0333 (5)	0.0610 (7)	−0.0055 (5)	−0.0394 (6)	−0.0006 (4)

### Geometric parameters (Å, º)

**Table:**

C1—N2	1.311 (2)	C11—N3	1.3166 (16)
C1—C2	1.4215 (19)	C11—C12	1.4309 (18)
C1—H1	0.9300	C11—C17	1.4890 (19)
C2—C3	1.399 (2)	C12—C16	1.4339 (18)
C2—C7	1.407 (2)	C12—C13	1.4394 (18)
C3—C4	1.3723 (19)	C13—O3	1.2575 (16)
C3—H3	0.9300	C13—C14	1.4447 (18)
C4—C5	1.408 (2)	C14—C15	1.325 (2)
C4—H4	0.9300	C14—H14	0.9300
C5—C6	1.3765 (18)	C15—O2	1.3649 (19)
C5—N3	1.4355 (15)	C15—C18	1.494 (2)
C6—C7	1.4022 (17)	C16—O1	1.2099 (19)
C6—H6	0.9300	C16—O2	1.4010 (18)
C7—N1	1.3672 (17)	C17—H17A	0.9600
C8—N1	1.456 (2)	C17—H17B	0.9600
C8—C9	1.495 (3)	C17—H17C	0.9600
C8—H8A	0.9700	C18—H18A	0.9600
C8—H8B	0.9700	C18—H18C	0.9600
C9—C10	1.284 (3)	C18—H18B	0.9600
C9—H9	0.9300	N1—N2	1.3684 (18)
C10—H10A	0.9300	N3—H3N	0.9090
C10—H10B	0.9300		
			
N2—C1—C2	111.83 (14)	C11—C12—C16	119.56 (12)
N2—C1—H1	124.1	C11—C12—C13	120.72 (11)
C2—C1—H1	124.1	C16—C12—C13	119.64 (12)
C3—C2—C7	119.78 (12)	O3—C13—C12	123.39 (12)
C3—C2—C1	136.29 (14)	O3—C13—C14	119.55 (12)
C7—C2—C1	103.93 (13)	C12—C13—C14	117.06 (12)
C4—C3—C2	118.11 (13)	C15—C14—C13	121.62 (13)
C4—C3—H3	120.9	C15—C14—H14	119.2
C2—C3—H3	120.9	C13—C14—H14	119.2
C3—C4—C5	121.11 (13)	C14—C15—O2	121.58 (13)
C3—C4—H4	119.4	C14—C15—C18	127.34 (16)
C5—C4—H4	119.4	O2—C15—C18	111.07 (14)
C6—C5—C4	122.61 (12)	O1—C16—O2	113.34 (13)
C6—C5—N3	120.24 (12)	O1—C16—C12	128.87 (14)
C4—C5—N3	116.93 (11)	O2—C16—C12	117.75 (13)
C5—C6—C7	115.68 (12)	C11—C17—H17A	109.5
C5—C6—H6	122.2	C11—C17—H17B	109.5
C7—C6—H6	122.2	H17A—C17—H17B	109.5
N1—C7—C6	130.43 (13)	C11—C17—H17C	109.5
N1—C7—C2	106.87 (12)	H17A—C17—H17C	109.5
C6—C7—C2	122.70 (12)	H17B—C17—H17C	109.5
N1—C8—C9	113.14 (15)	C15—C18—H18A	109.5
N1—C8—H8A	109.0	C15—C18—H18C	109.5
C9—C8—H8A	109.0	H18A—C18—H18C	109.5
N1—C8—H8B	109.0	C15—C18—H18B	109.5
C9—C8—H8B	109.0	H18A—C18—H18B	109.5
H8A—C8—H8B	107.8	H18C—C18—H18B	109.5
C10—C9—C8	126.33 (19)	C7—N1—N2	110.85 (13)
C10—C9—H9	116.8	C7—N1—C8	128.13 (13)
C8—C9—H9	116.8	N2—N1—C8	120.20 (12)
C9—C10—H10A	120.0	C1—N2—N1	106.50 (12)
C9—C10—H10B	120.0	C11—N3—C5	128.38 (11)
H10A—C10—H10B	120.0	C11—N3—H3N	113.9
N3—C11—C12	118.25 (11)	C5—N3—H3N	117.7
N3—C11—C17	118.15 (12)	C15—O2—C16	122.25 (11)
C12—C11—C17	123.55 (12)		

### Hydrogen-bond geometry (Å, º)

**Table:**

*D*—H···*A*	*D*—H	H···*A*	*D*···*A*	*D*—H···*A*
N3—H3*N*···O3	0.91	1.77	2.5567 (15)	143
N3—H3*N*···O3^i^	0.91	2.51	3.0612 (16)	120
C6—H6···O1^ii^	0.93	2.54	3.3554 (19)	146
C8—H8*A*···O1^ii^	0.97	2.53	3.495 (2)	173

Symmetry codes: (i) −*x*+1, −*y*, −*z*+1; (ii) −*x*+1, −*y*+1, −*z*+1.

## References

[bb1] Bruker (2009). *APEX2*, *SAINT* and *SADABS*. Bruker AXS Inc., Madison, Wisconsin, USA.

[bb2] Cerecetto, H., Gerpe, A., González, M., Arán, V. J. & de Ocáriz, C. O. (2005). *Mini Rev. Med. Chem.* **5**, 869–878. 10.2174/138955705774329564 16250831

[bb3] Farrugia, L. J. (2012). *J. Appl. Cryst.* **45**, 849–854.

[bb4] Jennings, A. & Tennant, M. (2007). *J. Chem. Inf. Model.* **47**, 1829–1838. 10.1021/ci600549q 17824684

[bb5] Paul, S., Panda, S. & Manna, D. (2014). *Tetrahedron Lett.* **55**, 2480–2483.

[bb6] Sheldrick, G. M. (2008). *Acta Cryst.* A**64**, 112–122. 10.1107/S0108767307043930 18156677

[bb7] Spek, A. L. (2009). *Acta Cryst.* D**65**, 148–155. 10.1107/S090744490804362X PMC2631630 19171970

[bb8] Sun, J. H., Teleha, C. A., Yan, J. S., Rodgers, J. D. & Nugiel, D. A. (1997). *J. Org. Chem.* **62**, 5627–5629.

[bb9] Westrip, S. P. (2010). *J. Appl. Cryst.* **43**, 920–925.

