# Supplementary figures and images for: Crystal structure of 3-{1-[(1-allyl-1H-indazol-6-yl)amino]­ethyl­idene}-6-methyl-2H-pyran-2,4(3H)-dione
Source: Acta Crystallogr Sect E Struct Rep Online. 2014 Nov 15;70(Pt 12):o1256. doi: 10.1107/S1600536814024520 (PMC4257434; doi:10.1107/S1600536814024520)

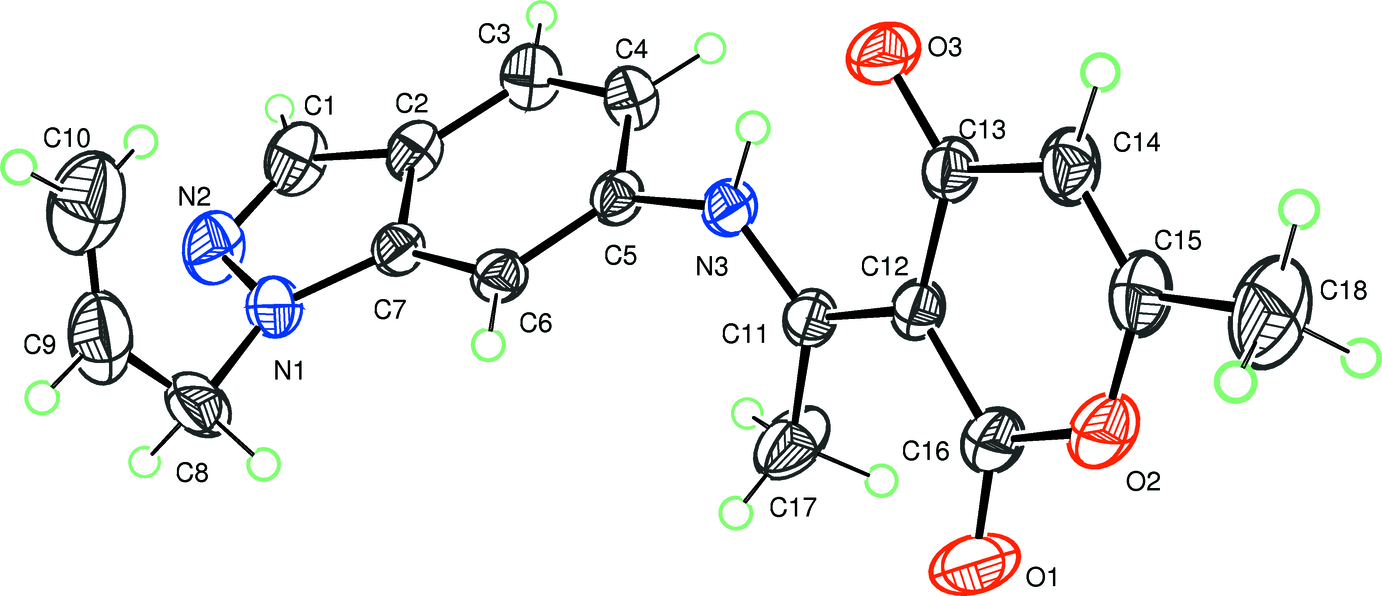

Supplement: Supplementary file 4 [file e-70-o1256-fig1.tif]

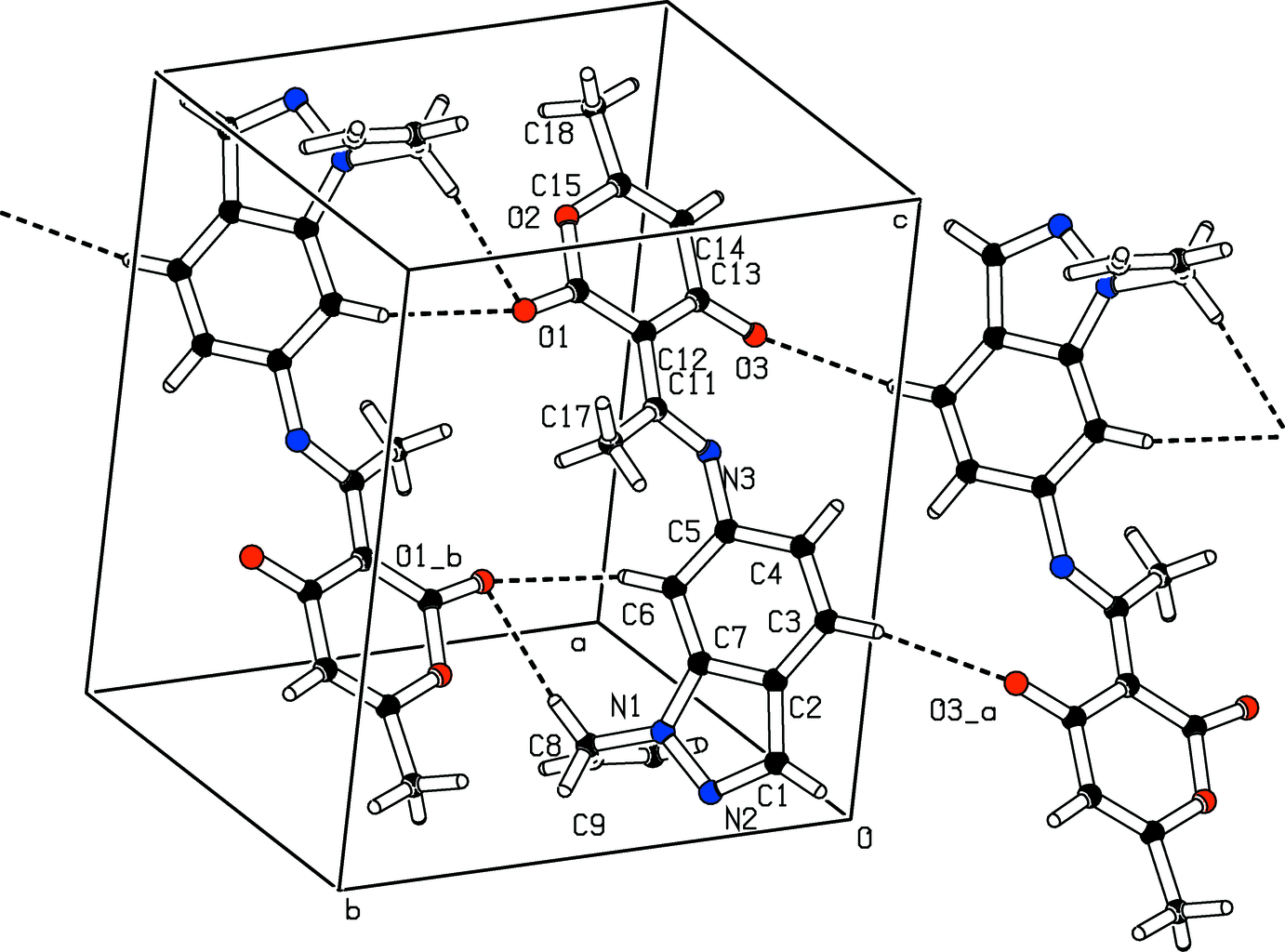

Supplement: Supplementary file 5 [file e-70-o1256-fig2.tif]
